# The association between systemic immune-inflammation index and rheumatoid arthritis: evidence from NHANES 1999–2018

**DOI:** 10.1186/s13075-023-03018-6

**Published:** 2023-03-04

**Authors:** Bo Liu, Jie Wang, Yan-yan Li, Kang-peng Li, Qiang Zhang

**Affiliations:** 1 Department of Orthopaedics, Beijing Ditan Hospital, Capital Medical University, Beijing, 100015 China; 2 Department of Integrated Traditional Chinese and Western Medicine, Beijing Ditan Hospital, Capital Medical University, Beijing, China

**Keywords:** Systemic immune-inflammation index, Rheumatoid arthritis, NHANES, Relationship, A cross-sectional study

## Abstract

**Purpose:**

We aimed to explore the relationship between the systemic immune-inflammation index (SII) and rheumatoid arthritis (RA) using NHANES from 1999 to 2018.

**Methods:**

We collected data from the NHANES database from 1999 to 2018. The SII is calculated from the counts of lymphocytes (LC), neutrophils (NC), and platelets (PC). The RA patients were derived from questionnaire data. We used weighted multivariate regression analysis and subgroup analysis to explore the relationship between SII and RA. Furthermore, the restricted cubic splines were used to explore the non-linear relationships.

**Result:**

Our study included a total of 37,604 patients, of which 2642 (7.03%) had rheumatoid arthritis. After adjusting for all covariates, the multivariate logistic regression analysis showed that high SII (In-transform) levels were associated with an increased likelihood of rheumatoid arthritis (OR=1.167, 95% CI=1.025–1.328, *P*=0.020). The interaction test revealed no significant effect on this connection. In the restricted cubic spline regression model, the relationship between ln-SII and RA was non-linear. The cutoff value of SII for RA was 578.25. The risk of rheumatoid arthritis increases rapidly when SII exceeds the cutoff value.

**Conclusion:**

In general, there is a positive correlation between SII and rheumatoid arthritis. Our study shows that SII is a novel, valuable, and convenient inflammatory marker that can be used to predict the risk of rheumatoid arthritis in US adults.

## Introduction

Rheumatoid arthritis (RA) is a chronic systemic inflammatory autoimmune disease characterized by diffuse polyarthritis and infiltration of pro-inflammatory cytokines [1, 2]. Joint fever, swelling and pain, pannus formation, and cartilage degeneration and bone erosion are the three classic pathological features of RA, as well as other systemic symptoms outside the joints, such as rash, fever, muscle loss, and weakness [3, 4]. RA is more common in women in their 40s. Previous studies have shown that the pathogenesis of rheumatoid arthritis is complex. At the cellular level, it is mainly manifested by an imbalance between osteoblasts and osteoclasts, excessive proliferation of synoviocytes, and immune cell dysfunction [5]. The cellular inflammatory factors can also lead to rheumatoid arthritis, such as interleukin (IL)-17, tumor necrosis factor (TNF-α), IL-6, and IL-8 [6, 7]. Recent studies have shown that the emergence of autoreactive T cells is a crucial pathological event in patients with RA [7]. Primitive CD4 T cells differentiate into pro-inflammatory helper T cells, which are more easily able to invade tissues and cause inflammation through immune cell death [7–9].

Systemic immune-inflammation index (Sll), as an evaluation index of systemic inflammatory response, has been confirmed to be related to the prognosis of elderly patients with digestive system tumors [10, 11]. It is calculated using a formula that takes into account the levels of certain immune system markers in the blood, The calculation formula is platelets × neutrophils ÷ lymphocytes [11]. The SII is often used as a predictor of mortality in patients with tumors, as higher SII values have been associated with an increased risk of death [10, 12, 13]. This may be related to the imbalance between the body’s tumor-promoting and anti-tumor factors in the tumor state. When the imbalance occurs, neutrophils and platelets increase, lymphocytes decrease, and the level of SII also increases. In recent years, the application field of SII has been expanding continuously, and more studies have shown that SII can also be used to predict the severity of certain diseases and monitor treatment effects [14–18].

The systemic immune-inflammatory index (SII) may be useful in assessing the severity and progression of psoriasis and psoriatic arthritis (PA). In people with higher SII levels, the immune system is in a constant state of activation, leading to chronic inflammation in the joints and other tissues [19, 20]. This inflammation can damage cartilage and bone, causing pain and difficulty moving. Satis, S. et al. [21] found that the SII levels were significantly higher in patients with rheumatoid arthritis compared to healthy controls and that SII were correlated with the severity of the disease. These findings suggest that the SII may be a useful tool for monitoring inflammation and disease activity in patients with RA.

The National Health and Nutrition Examination Survey (NHANES) is a population-based cross-sectional survey designed to collect information about the health and nutrition of American households. The database uses a complex stratified, multistage probability cluster sampling design to represent the entire US population [22]. However, to date, no researchers have used the NHANES database to explore the relationship between SII and RA. Our study aimed to clarify the relationship between SII and RA in participants of the NHANES. We hypothesized that the RA patients have higher SII.

## Methods

### Data selection and study design

We download data from the NHANES database, which surveys about 5000 individuals from across the country each year. The database includes demographic data, dietary data, examination data, laboratory data, questionnaire data, and limited access data. NHANES was conducted over 10 cycles from 1999 to 2018. This research received approval from the National Center for Health Statistics Research Ethics Review Board, and participants signed informed consent forms. The detailed NHANES study design and data are publicly available at https://www.cdc.gov/nchs/nhanes/.

**Fig. 1:**
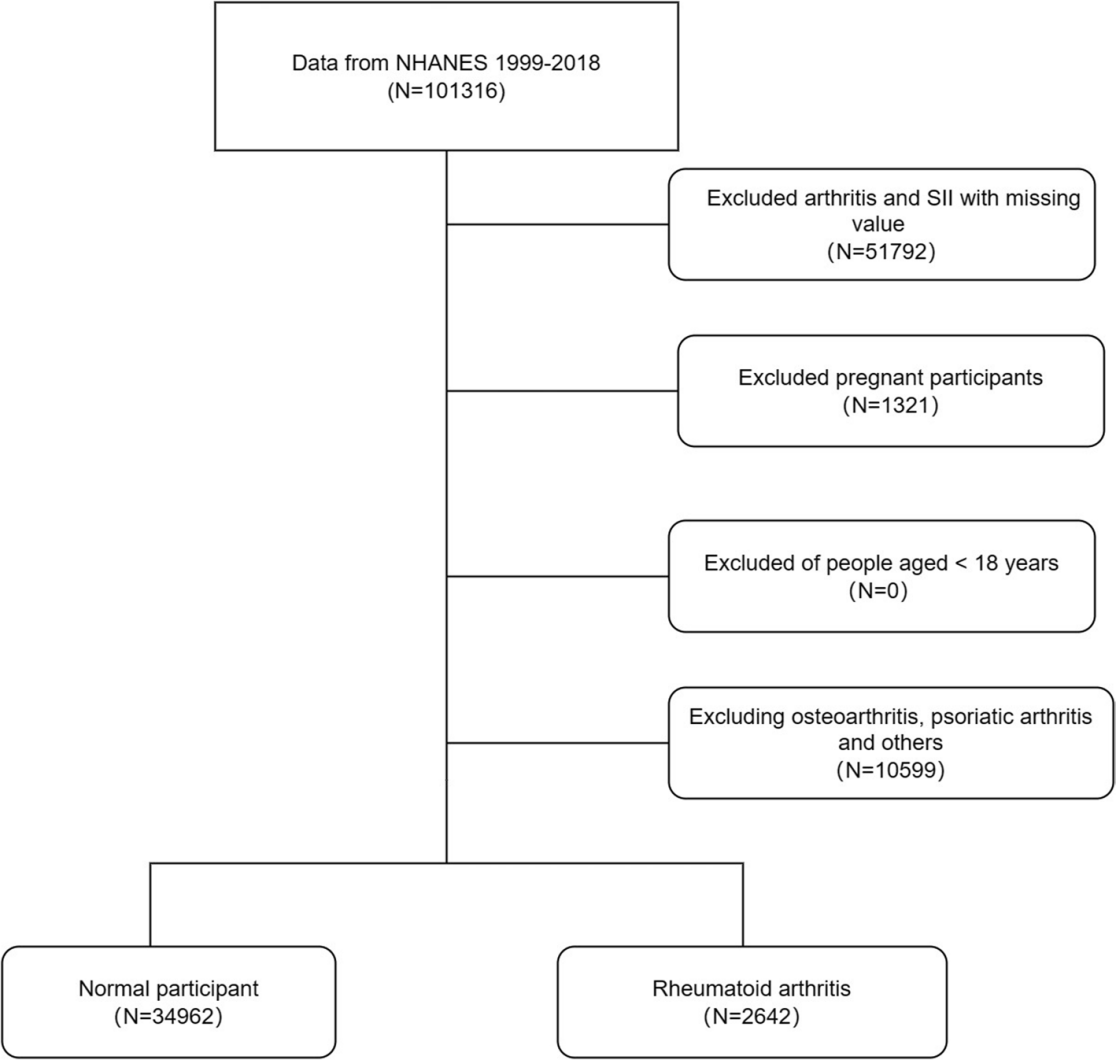
Flowchart of the participant selection from NHANES 1999–2018

Our study exclusions were as follows: (1) adults aged 18 years or older; (2) pregnant women; (3) individuals with missing data on arthritis; (4) individuals with missing data on platelets, neutrophils, and lymphocytes (Fig. 1). A total of 37,604 individuals were ultimately included in this study. Considering our study included hematology variables, we chose Mobile Examination Centers (MEC) weights. The weight calculation formula for 1999–2000 and 2001–2002 was 2/10 × wtmec4yr, and the weight calculation formula for 2003–2018 was 1/10 × wtmec2yr.

### The definition of systemic immune-inflammation index

**Fig. 2:**
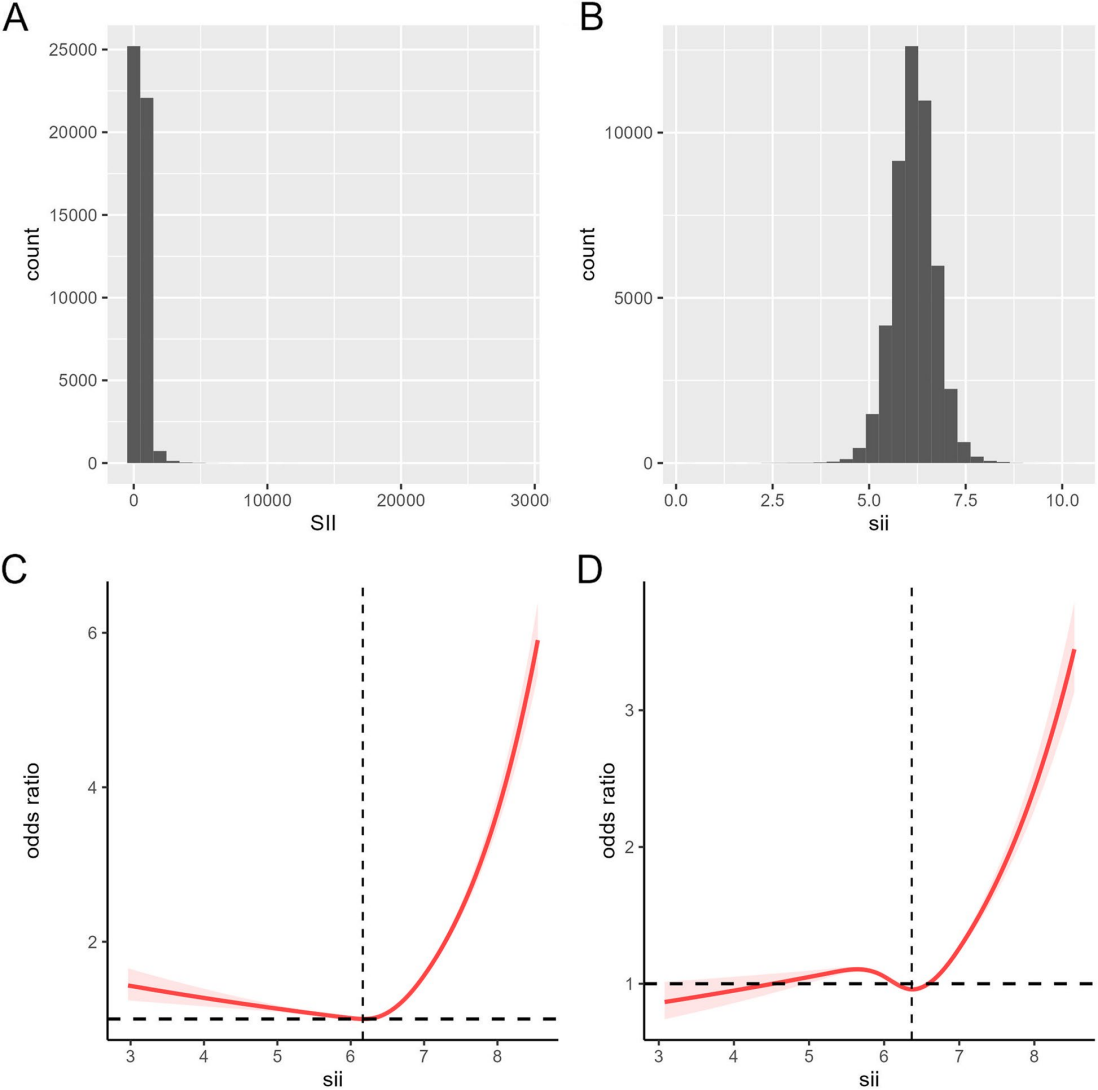
The distribution of SII (**A**). The distribution of ln-transformed SII (**B**). The non-adjusted relationship between SII and RA (**C**). The full-adjusted relationship between SII and IA (**D**)

The methods used to derive CBC parameters are based on the Beckman Coulter methodology of counting and sizing, in combination with an automatic diluting and mixing device for sample processing, and a single beam photometer for hemoglobinometry. The WBC differential uses VCS technology. The Beckman Coulter DxH 800 instrument in the NHANES mobile examination center (MEC) produces a CBC on blood specimens and provides a distribution of blood cells for all participants. According to previous research reports, the calculation formula of SII is platelet count × neutrophil count/lymphocyte count [11, 18]. In addition, SII was log2-transformed when conducting regression analysis (Fig. 2B), considering that these inflammatory markers were right-skewed distributed (Fig. 2A).

### The definition of rheumatoid arthritis

The diagnosis of arthritis was obtained by a self-report questionnaire (MCQ160a). Specifically, participants were asked that “Has a doctor or other health professional ever told you that you had arthritis?” The response options were “Yes” or “No.” Rheumatoid arthritis was assessed via the following question: “Which type of arthritis was it?” The response options were “Rheumatoid arthritis,” Osteoarthritis,” “Psoriatic arthritis,” “Other,” “Refused,” and “Don’t know.” A previous study demonstrated great consistency (85%) between self-reported arthritis and clinically confirmed arthritis [23].

### The covariates

The covariates included in our study that may affect the RA include age (< 40, 40–59, ≥60), gender (male, female), race (White, Black, Mexican American, other), education (under high school, high school or equivalent, college graduate or above), poverty-to-income ratio (PIR<1.3, 1.3–3.49, >= 3.5), body mass index (<25, 25–29.9, ≥30), work activity (yes, no), smoking (never, former, current), alcohol use (never, former, mild, moderate, heavy), hypertension (yes, no), diabetes (no, pre-diabetes, diabetes), hyperlipidemia (yes, no), and complete blood count (white blood cells, red blood cells).

### Statistical methods

All analyzes were performed using R (version 4.1.3, http://www.R-project.org). The NHANES database was surveyed using complex, multi-stage, sampling, therefore, our study used MEC exam weight (WTMEC4YR, WTMEC2YR) for analysis. Continuous variables are presented as weighted means and standard deviations, and categorical variables are presented as weighted percentages. We compared categorical variables and continuous variables between different groups using the chi-square test and *T*-test, respectively.

We found that the SII data is unevenly distributed and clearly skewed to the right. Therefore, prior to conducting statistical analysis, we need to ln-transform its values. We analyzed the association of RA and SII using weighted multivariate logistic regression models. In the crude model, covariates were not adjusted. In model 1, gender, age, and race were adjusted. In model 2, age, gender, race, PIR, edu, and BMI were adjusted. Model 3 was adjusted for age, gender, race, PIR, edu, BMI, hypertension, diabetes, hyperlipidemia, alcohol user, smoke, work activity, white blood cell count, and red blood cell count. Furthermore, we considered the systemic immunity index as a categorical variable by Quartile. The restricted cubic splines were used to explore the non-linear relationships. To explore the threshold effect of In-SII on the risk of rheumatoid arthritis and to find the inflection point, we used the smooth curve fitting and generalized additive models.

Finally, we further performed stratification and interaction analyses by age, PIR, BMI, race, edu, hypertension, diabetes, hyperlipidemia, work activity, alcohol user, and smoke. All statistical tests were two-sided, and a *P*-value < 0.05 was statistically significant.

## Results

### General characteristics of the study population

A total of 36,463 people were included in this study, of whom 51.9% were male and 48.1% were female, 26.8% were over 60 years of age and 41.0% were white. The number of patients diagnosed with rheumatoid arthritis was 2642 (7.0%). The clinical characteristics of the participants by SII quartiles are shown in Table 1, from which we can find statistically significant differences in age, gender, race, education, BMI, hypertension, diabetes, hyperlipidemia, smoking, alcohol use, and work activity (all *p*<0.05).

**Table 1:** Weighted demographic characteristics of all participants

Variable	Total	Q1	Q2	Q3	Q4	*P*-value
SII	6.181 (0.005)	5.507 (0.004)	5.994 (0.001)	6.316 (0.001)	6.806 (0.004)	< 0.0001
Gender						< 0.0001
Male	19,523 (51.917)	5399 (57.462)	5001 (53.229)	4703 (50.380)	4420 (44.756)	
Female	18,081 (48.083)	4015 (42.538)	4391 (46.771)	4691 (49.620)	4984 (55.244)	
Age						< 0.0001
Below 60	27,516 (73.173)	6892 (82.354)	6949 (82.953)	7032 (82.786)	6643 (80.032)	
Over 60	10,088 (26.827)	2522 (17.646)	2443 (17.047)	2362 (17.214)	2761 (19.968)	
Race						< 0.0001
White	15,436 (41.049)	2842 (56.742)	3777 (65.819)	4222 (68.871)	4595 (71.053)	
Black	7901 (21.011)	3177 (19.964)	1845 (10.402)	1497 ( 8.327)	1382 ( 7.603)	
Mexican American	7178 (19.088)	1497 (8.905)	1860 (9.461)	1948 (9.664)	1873 (9.001)	
Other	7089 (18.852)	1898 (14.389)	1910 (14.318)	1727 (13.138)	1554 (12.344)	
Edu						< 0.0001
Under high school	9920 (26.414)	2506 (17.438)	2468 (16.382)	2460 (16.301)	2486 (16.818)	
High school or equivalent	8561 (22.795)	2063 (22.092)	2048 (22.397)	2177 (24.450)	2273 (25.640)	
College graduate or above	19,075 (50.791)	4833 (60.469)	4865 (61.221)	4744 (59.249)	4633 (57.542)	
PIR						0.055
Below 1.3	10,545 (30.684)	2659 (21.958)	2571 (20.555)	2602 (20.699)	2713 (22.376)	
1.3–3.5	13,049 (37.97)	3261 (35.950)	3247 (35.517)	3237 (35.238)	3304 (36.065)	
Over 3.5	10,773 (31.347)	2631 (42.091)	2767 (43.928)	2761 (44.064)	2614 (41.560)	
BMI						< 0.0001
Below 25	11,841 (31.975)	3218 (37.163)	2977 (34.391)	2766 (30.822)	2880 (32.605)	
25–30	12,783 (34.519)	3316 (34.919)	3298 (35.603)	3244 (34.543)	2925 (31.384)	
Over 30	12,408 (33.506)	2772 (27.918)	3013 (30.006)	3243 (34.635)	3380 (36.010)	
Hypertension						< 0.0001
No	23,543 (62.608)	6017 (69.978)	6052 (70.143)	5923 (67.733)	5551 (64.058)	
Yes	14,061 (37.392)	3397 (30.022)	3340 (29.857)	3471 (32.267)	3853 (35.942)	
Diabetes						< 0.0001
No	29,412 (78.215)	7459 (83.957)	7362 (83.934)	7433 (83.852)	7158 (81.057)	
Pre-diabetes	2523 (6.709)	596 (6.101)	627 (6.013)	639 (6.282)	661 (6.713)	
Diabetes	5669 (15.076)	1359 ( 9.942)	1403 (10.052)	1322 ( 9.866)	1585 (12.230)	
Hyperlipidemia						< 0.0001
No	11,672 (31.039)	3321 (37.126)	2899 (32.367)	2688 (29.487)	2764 (30.289)	
Yes	25,932 (68.961)	6093 (62.874)	6493 (67.633)	6706 (70.513)	6640 (69.711)	
Alcohol use						< 0.0001
Never	4805 (14.232)	1241 (12.196)	1217 (11.216)	1147 (10.663)	1200 (11.052)	
Former	5315 (15.743)	1282 (11.817)	1246 (11.648)	1313 (13.312)	1474 (14.559)	
Mild	10,961 (32.465)	2905 (36.822)	2774 (36.178)	2711 (34.791)	2571 (31.805)	
Moderate	5191 (15.375)	1256 (17.608)	1332 (17.717)	1319 (17.393)	1284 (17.206)	
Heavy	7490 (22.185)	1691 (21.557)	1836 (23.241)	1982 (23.841)	1981 (25.377)	
Smoke						< 0.0001
Never	21,053 (56.031)	5508 (57.649)	5391 (57.413)	5271 (55.789)	4883 (51.418)	
Former	8346 (22.212)	2023 (22.562)	2081 (22.149)	2010 (21.402)	2232 (23.299)	
Now	8175 (21.757)	1874 (19.789)	1912 (20.439)	2108 (22.809)	2281 (25.283)	
Work activity						0.006
No	19,774 (52.595)	5159 (48.399)	4875 (45.917)	4831 (44.956)	4909 (46.196)	
Yes	17,823 (47.405)	4255 (51.601)	4513 (54.083)	4563 (55.044)	4492 (53.804)	
Arthritis						< 0.0001
No	34,962 (92.974)	8806 (94.972)	8803 (95.222)	8780 (95.295)	8573 (93.514)	
Yes	2642 (7.026)	608 (5.028)	589 (4.778)	614 (4.705)	831 (6.486)	
White blood cell	7.235 (0.025)	6.139 (0.039)	6.750 (0.025)	7.357 (0.029)	8.526 (0.035)	< 0.0001
Red blood cell	4.736 (0.006)	4.717 (0.008)	4.747 (0.009)	4.755 (0.007)	4.724 (0.009)	< 0.0001
Lymphocyte	2.151 (0.009)	2.456 (0.026)	2.220 (0.010)	2.094 (0.010)	1.877 (0.009)	< 0.0001
Neutrophils	4.281 (0.018)	2.932 (0.015)	3.747 (0.015)	4.448 (0.018)	5.791 (0.027)	< 0.0001
Platelet	254.407 (0.655)	209.898 (0.780)	240.372 (0.796)	263.130 (0.816)	297.571 (1.011)	< 0.0001

### Univariate logistic regression analysis of RA

Based on Table 2, it can be concluded that the risk of rheumatoid arthritis is increased (OR > 1, *p* < 0.05) in individuals with older age (> 40 years), female, black race, high BMI (> 25), smoking, diabetes (yes), hypertension (yes), and hyperlipidemia (yes). However, participants who were Mexican-Americans, other races, PIR (> 1.3), higher education, alcohol use, and work activities (yes) show a reduced risk of rheumatoid arthritis (OR < 1, *p* < 0.05).

**Table 2:** Weighted univariate logistic analysis of RA

Character	OR 95% CI	*P*-value
Age		
Below 60	ref	ref
Over 60	4.501 (4.024, 5.034)	<0.0001
BMI		
Below 25	ref	ref
25–30	1.224 (1.064, 1.409)	0.005
Over 30	2.018 (1.758, 2.317)	<0.0001
PIR		
Below 1.3	ref	ref
1.3–3.5	0.698 (0.601, 0.810)	<0.0001
Over 3.5	0.476 (0.406, 0.557)	<0.0001
Gender		
Male	ref	ref
Female	1.545 (1.377, 1.733)	<0.0001
Race		
White	ref	ref
Black	1.485 (1.307, 1.687)	<0.0001
Mexican American	0.684 (0.580, 0.806)	<0.0001
Other	0.760 (0.633, 0.912)	0.003
Edu		
Under high school	ref	ref
High school or equivalent	0.719 (0.631, 0.818)	<0.0001
College graduate or above	0.453 (0.391, 0.524)	<0.0001
Hypertension		
No	ref	ref
Yes	3.925 (3.461, 4.450)	<0.0001
Diabetes		
No	ref	ref
Pre-diabetes	1.753 (1.474, 2.084)	<0.0001
Diabetes	3.043 (2.711, 3.416)	<0.0001
Hyperlipidemia		
No	ref	ref
Yes	2.041 (1.770, 2.354)	<0.0001
Alcohol use		
Never	ref	ref
Former	1.589 (1.341, 1.882)	<0.0001
Mild	0.730 (0.612, 0.869)	<0.001
Moderate	0.557 (0.453, 0.684)	<0.0001
Heavy	0.511 (0.420, 0.621)	<0.0001
Smoke		
Never	ref	ref
Former	1.968 (1.704, 2.272)	<0.0001
Now	1.626 (1.408, 1.878)	<0.0001
Work activity		
No	ref	ref
Yes	0.753 (0.678, 0.837)	<0.0001
SII	1.296 (1.166, 1.441)	<0.0001

### Relationship between RA and SII

After performing a weighted multivariate logistic regression analysis (Table 3), our results indicate that a higher SII score is associated with an increased risk of developing rheumatoid arthritis. This association was significant in our crude model (OR=1.296; 95% CI=1.166–1.441, *p*<0.001), model 1 (OR=1.291; 95% CI=1.164–1.433, *p*<0.001), and model 2 (OR=1.192; 95% CI=1.066–1.332, *p*=0.002). In the fully adjusted model, the positive association between SII and proteinuria remained stable (OR=1.167; 95% CI=1.025–1.328, *p*=0.020), indicating that for every unit increase in In-SII score, the risk of developing rheumatoid arthritis increased by 17%.

**Table 3:** Weighted multivariate logistic analysis systemic immune-inflammation index and rheumatoid arthritis

	Crude model		Model 1		Model 2		Model 3	
	OR 95% CI	*P*-value	OR 95% CI	*P*-value	OR 95% CI	*P*-value	OR 95% CI	*P*-value
SII	1.296 (1.166, 1.441)	<0.001	1.291 (1.164, 1.433)	<0.001	1.192 (1.066, 1.332)	0.002	1.167 (1.025, 1.328)	0.020
**Stratified by SII quartiles**								
Q1	ref		ref		ref		ref	
Q2	0.948 (0.818, 1.098)	0.474	1.003 (0.857, 1.173)	0.973	0.980 (0.826, 1.162)	0.811	0.975 (0.806, 1.181)	0.796
Q3	0.933 (0.789, 1.102)	0.411	0.991 (0.832, 1.180)	0.915	0.940 (0.787, 1.124)	0.498	0.906 (0.745, 1.102)	0.320
Q4	1.310 (1.128, 1.521)	<0.001	1.323 (1.127, 1.554)	<0.001	1.200 (1.013, 1.421)	0.035	1.138 (0.933, 1.388)	0.201
*P* for trend		<0.001		<0.001		0.038		0.254

We further transformed the SII from a continuous variable into a categorical variable (quartiles) for sensitivity analysis (Table 3). Compared with the lowest quartile, the risk of developing rheumatoid arthritis in the highest quartile increased by 31% (OR=1.310; 95% CI=1.128–1.521, *p*<0.001) in the crude model, 32% (OR=1.323; 95% CI=1.127–1.554, *p*<0.001) in the model 1 and 20% (OR=1.200; 95% CI=1.013–1.421, *p*=0.035).

### The non-linear relationship between RA and SII

Using restricted cubic splines, a non-linear relationship between ln-SII and RA risk was found in the original model (Fig. 2C) and after adjustment for multiple covariates (Fig. 2D) (*p*<0.001). Moreover, A threshold effect can be observed, with an inflection point at the ln-SII value of 6.36 (SII = 578.25). when the SII value is less than the cutoff value, the risk of rheumatoid arthritis is almost unchanged or even decreased, and when the SII value exceeds the cutoff value, the risk increases rapidly.

### The subgroup analysis and interaction test

We found that the risk of rheumatoid arthritis was not consistently associated with increased SII levels (Table 4) in some subgroups. Overall, for PIR (>3.5), participants with diabetes, alcohol use (moderate) and smoking, this correlation was not statistically significant (*P*>0.05).

**Table 4:** Subgroup analysis for the association between SII and arthritis

Character	OR 95% CI	*P*-value	*P* for interaction
Age			0.795
Below 60	1.253 (1.062, 1.479)	0.008	
Over 60	1.355 (1.196, 1.535)	<0.0001	
PIR			0.318
Below 1.3	1.200 (1.012, 1.422)	0.036	
1.3–3.5	1.402 (1.191, 1.651)	<0.0001	
Over 3.5	1.173 (0.929, 1.482)	0.178	
BMI			0.25
Below 25	1.392 (1.118, 1.732)	0.003	
25–30	1.324 (1.059, 1.655)	0.014	
Over 30	1.172 (1.011, 1.358)	0.035	
Gender			0.051
Male	1.428 (1.203, 1.695)	<0.0001	
Female	1.173 (1.011, 1.361)	0.035	
Hypertension			0.645
No	1.177 (0.972, 1.425)	0.095	
Yes	1.272 (1.121, 1.443)	<0.001	
Diabetes			0.08
No	1.320 (1.144, 1.524)	<0.001	
Pre-diabetes	1.513 (1.092, 2.096)	0.013	
Diabetes	1.091 (0.905, 1.314)	0.359	
Hyperlipidemia			0.111
No	1.583 (1.221, 2.053)	<0.001	
Yes	1.230 (1.092, 1.385)	<0.001	
Work activity			0.497
No	1.361 (1.162, 1.593)	<0.001	
Yes	1.263 (1.082, 1.475)	0.003	
Alcohol use			0.405
Never	1.581 (1.229, 2.035)	<0.001	
Former	1.266 (1.072, 1.496)	0.006	
Mild	1.250 (1.007, 1.552)	0.044	
Moderate	1.140 (0.851, 1.528)	0.377	
Heavy	1.409 (1.016, 1.954)	0.040	
Smoke			0.643
Never	1.342 (1.118, 1.611)	0.002	
Former	1.408 (1.150, 1.724)	0.001	
Now	1.171 (0.964, 1.422)	0.112	

Furthermore, the interaction test showed that gender, age, BMI, PIR, hypertension, diabetes, hyperlipidemia, smoking, alcohol use, and work activity had no significant effect on this connection (Table 4, interaction all *P*>0.05).

## Discussion

This study finally included 37,604 participants from the NHANES 1999–2018 cohort for analysis, including 19,523 males and 18,081 females. Of these, 2642 patients had rheumatoid arthritis. Compared with normal people, patients with RA have higher levels of SII. Moreover, after adjusting for all covariates, we found that the relationship was non-linear. Further, we found that when SII is higher than 578.25, the risk of rheumatoid arthritis will increase significantly. And there were stratification effects in the PIR of more than 3.5, diabetes, alcohol moderate use, and smoking population.

To the best of our knowledge, our study is the first to report that the SII level of patients with RA was higher than that of healthy controls by using the NHANES database. Our results are consistent with previous research. Choe et al [24] suggested that the SII scores may be useful markers that adequately reflect the activity of the RA and may lead to more accurate diagnoses. Satis et al. [21] found that the systemic immune-inflammation index could be used as a new tool, showing the RA disease activity, and through the ROC curve, they concluded that 574.20 is the best cut-off point for active RA. Their study found that the cutoff values are roughly similar to those in our study. This shows that this point has certain clinical value. However, that study also has certain limitations. For example, the study was a case-control study with a low sample size.

Compared with previous studies, our conclusions are more convincing and provide sufficient evidence. The study by Kelesoglu et al. [19] collected 106 psoriatic arthritis (PsA) patients and 103 age and gender-matched healthy individuals, showing that compared with patients in remission or with low disease activity, SII levels were significantly higher in PsA patients with moderate to severe disease (*p*<0.001). Similarly, Yorulmaz et al. [20] also demonstrated that SII might serve as an independent prognostic indicator for patients with psoriasis and psoriatic arthritis. Wu et al. [25] also confirmed that for patients with ankylosing spondylitis, SII was increased in AS. The SII may be a novel indicator for monitoring disease activity in AS.

Overall, there are few studies about the systemic immunoinflammatory index and rheumatoid arthritis. Moreover, researchers must pay more attention to the fact that the systemic immune-inflammation index is a right-skewed non-normal distribution. Before the data analysis process, it is better to perform log transformation. The systemic immune-inflammation index (SII) measures the level of systemic inflammation in patients with RA and has been shown to be a strong predictor of disease activity, joint damage, and radiographic progression. In general, higher SII values are associated with more severe RA disease activity and a poorer prognosis.

Rheumatoid arthritis affects about 0.5 to 1% of adults in developed countries, with about 5 to 50 new cases per 100,000 people each year [26]. The disease most commonly occurs in middle age, and the incidence rate in women is 2.5 times higher than in men [27, 28]. Rheumatoid arthritis caused 28,000 deaths in 1990 and 38,000 deaths in 2013 [29]. Rheumatoid arthritis (RA) is a chronic autoimmune disease characterized by inflammation of the joints, leading to pain, swelling, stiffness, and progressive joint damage. RA is a systemic disease that affects multiple joints and other organ systems, including the skin, eyes, lungs, and cardiovascular system [28]. RA is also associated with extra-articular manifestations such as rheumatoid nodules, anemia and fatigue. Osteoarthritis and rheumatoid arthritis are two completely different diseases. Osteoarthritis is cartilage degeneration [30]; rheumatoid arthritis is a systemic autoimmune disease.

The exact cause of rheumatoid arthritis (RA) is unknown, but it is believed to be a complex interaction of genetic, environmental, and immunologic factors. There is evidence of genetic predisposition to the development of RA, with certain HLA alleles being associated with increased susceptibility. Environmental factors, such as infections, trauma, and smoking, have also been implicated in the etiology of RA [4]. The immune system of RA patients is characterized by systemic inflammation and autoantibody production, leading to the activation of immune cells and the release that contribute to joint damage and functional impairment [31]. While the exact cause of RA remains unclear, a better understanding of the underlying mechanisms may lead to the development of new and effective therapies for this debilitating disease.

The systemic immune-inflammation index (SII) is an indicator used to assess the degree of systemic inflammation in an individual. It is calculated by the platelet count, neutrophil count, and lymphocyte count (PLT×N/L ratio) [11]. Overall, some researchers found that this indicator could be used to predict mortality in cancer patients. Other researchers think it can be used as an indicator of the progression of a certain disease. The study by Li et al. [13] suggested that high SII levels may increase overall mortality and cardiovascular disease mortality in the general population, using NHANES follow-up data from 1999 to 2014. He et al. [12] showed a correlation between SII and all-cause mortality in the US arteriosclerotic cardiovascular disease (ASCVD) population. Increased SII was associated with poor survival in ASCVD patients. Guo et al. [32] found that SII levels in T2DM patients were associated with the development of diabetic kidney disease (DKD). SII may be a cost-effective and simple method to detect DKD. Zhang et al. [33] have shown that elevated levels of SII may be a potential risk factor for the development of osteoporosis in post-menopausal women. A similar conclusion was reached by Tang et al. [17].

Our research has some advantages. This is the first study to explore the relationship between the systemic immune-inflammation index and osteoarthritis based on the large sample size of the NHANES database. Furthermore, we use a weighted logistic regression model for analysis since the NHANES database is composed of multi-stage complex sampling data, and we have adjusted other covariates, which makes the conclusions drawn in this study more accurate and reliable. Additionally, we ln-transformed the SII before the analysis process to ensure it had a normal distribution. Finally, we used restrictive cubic spline and smooth curve fitting to explore their non-linear relationship and further calculated the inflection points.

However, our study also has some limitations. First, some variables in this study come from questionnaires and self-reports, which are prone to bias. Furthermore, since NHANES did not record some classic inflammatory factors (such as TNF-α, interleukin-6, interleukin-10, etc.), relevant indicators cannot be included to obtain more comprehensive results. More researchers should continue to explore the inflammatory markers in rheumatoid arthritis. We hope this study provides a scientific data reference for future research. We will also include more classical inflammatory factors in future studies to explore the relationship between osteoarthritis and systemic immune-inflammatory indices.

## Conclusion

In summary, our study provides new insights into the relationship between rheumatoid arthritis and SII. In general, there is a positive correlation between SII and rheumatoid arthritis. The SII cut-off (578.25) has a certain clinical application value. Our study shows that SII is a novel, valuable, and convenient inflammatory marker that can be used to predict the risk of rheumatoid arthritis in adults. Low cost and easy to collect and calculate are the advantages of SII. We hope that the SII will become a good evaluation index for predicting cancer prognosis and evaluating disease activity.

## Data Availability

Publicly available datasets were analyzed in this study. This data can be found here: The National Health and Nutrition Examination Survey dataset at https://www.cdc.gov/nchs/nhanes/index.htm

## Abbreviations

SII: Systemic immune-inflammation index
RA: Rheumatoid arthritis
OA: Osteoarthritis
PA: Psoriatic arthritis
AS: Ankylosing spondylitis
NHANES: National Health and Nutrition Examination Survey
BMI: Body mass index
CI: Confidence interval
